# Improved Genome Editing in Human Cell Lines Using the CRISPR Method

**DOI:** 10.1371/journal.pone.0109752

**Published:** 2014-10-10

**Authors:** Ivan M. Munoz, Piotr Szyniarowski, Rachel Toth, John Rouse, Christophe Lachaud

**Affiliations:** Medical Research Council Protein Phosphorylation Unit, College of Life Sciences, University of Dundee, Dundee, Scotland; NIH, United States of America

## Abstract

The Cas9/CRISPR system has become a popular choice for genome editing. In this system, binding of a single guide (sg) RNA to a cognate genomic sequence enables the Cas9 nuclease to induce a double-strand break at that locus. This break is next repaired by an error-prone mechanism, leading to mutation and gene disruption. In this study we describe a range of refinements of the method, including stable cell lines expressing Cas9, and a PCR based protocol for the generation of the sgRNA. We also describe a simple methodology that allows both elimination of Cas9 from cells after gene disruption and re-introduction of the disrupted gene. This advance enables easy assessment of the off target effects associated with gene disruption, as well as phenotype-based structure-function analysis. In our study, we used the Fan1 DNA repair gene as control in these experiments. Cas9/CRISPR-mediated Fan1 disruption occurred at frequencies of around 29%, and resulted in the anticipated spectrum of genotoxin hypersensitivity, which was rescued by re-introduction of Fan1.

## Introduction

Technologies enabling gene knockout in somatic cells are vital for delineating gene function. A range of such technologies based on nucleases have been developed in recent years [1], [2], [3], [4]. The most recent genome-editing technology employs the Cas9/CRISPR (clustered regularly interspaced short palindromic repeats) nuclease from *Streptococcus pyogenes*, which acts as a form of innate immunity in bacteria. This system employs a single guide (sg) RNA which has two main features: a domain which targets a specific genomic DNA sequence (targeting domain) and a scaffold domain which binds the Cas9 nuclease [5], [6]. Although the mechanism underlying the ability of the sgRNA to find its target sequence is not clear, it is known that target recognition requires both sequence complementarity as well as a specific tri-nucleotide motif, referred to as the “protospacer-adjacent motif (PAM)” sequence, at the 3′ end of the target sequence. Binding of the sgRNA to the cognate sequence in the human genome recruits the Cas9 nuclease, enabling the generation of a double strand break (DSB) within the target gene. Repair of the resulting DSB by non-homologous end-joining (NHEJ) often leads to small deletions or insertions that, in the coding region of a gene, can result in frame-shifting and, therefore, gene disruption [7].

A concern with the Cas9/CRISPR system is the potential for off target effects. The sgRNA-Cas9 complexes are in general tolerant of 1–3 mismatches in their target DNA sequence, raising the possibility of disruption of non-specific target genes [8]. Steps can be taken to reduce the likelihood of off-target effects [9], [10], [11], [12] but these extra steps increase the time taken to effect gene disruption, and it is not clear that off-target effects are completely eliminated. Another concern is the persistence of the Cas9 nuclease in the gene-disrupted cells, and the potential consequences for cell function.

In this study we describe a range of refinements that combine to dramatically increase the ease and efficiency of gene disruption using the Cas9/CRISPR system. We go on to describe simple methodology that allows Cas9 to be eliminated from cells after gene disruption, and the disrupted gene to be re-introduced. This advance enables easy assessment of the off target effects associated with gene disruption, and also enables phenotype-based structure-function analysis.

## Materials and Methods

### Construction of sgRNA coding vector (pEsgRNA)

The sequence corresponding to the U6-BbsI-chiRNA from the Addgene 45946 vector was chemically synthetized and inserted into the SfiI site of a pMA-T vector (Invitrogen). The BbSI site was then mutated by PCR into a BamHI site using the following primers:

5′-GGAAAGGACGAAACACCGGGTCTGGATCCGACCTGTTTTAGAGCTAGAAAT-3′ 5′-ATTTCTAGCTCTAAAACAGGTCGGATCCAGACCCGGTGTTTCGTCCTTTCC-3′

### Generation of the sgRNA plasmid

We designed a vector (pEsgRNA) that has a U6 promoter element upstream of an element encoding the scaffold domain of the sgRNA (Figure S1). The objective was to use insertion-based mutagenesis to introduce a gene-specific targeting domain between U6 and the scaffold domain. This would enable U6-directed expression of the reconstituted sgRNA without the need for conventional cloning. Insertion mutagenesis relied on primers containing 20 nucleotides corresponding to the targeted domain, flanked by sequences complementary to motifs that straddle a BamHI site between the U6 and scaffold domain in vector pEsgRNA (Figure S1). PCR reactions with these primers enabled the generation of the complete and functional sgRNA vector. To remove template plasmid with no insertion, the PCR reaction was digested with DpnI, which only cleaves methylated DNA (Figure S1). The pEsgRNA parent plasmid has two BamHI sites: one in the U6 element and one between the U6 and scaffold domain. Successful mutagenesis results in loss of the latter site, so that BamHI digestion cleaves once, not twice. After insertion mutagenesis, plasmids were digested with BamHI and those plasmids that had lost the second BamHI site were cloned and sent for sequencing to confirm insertion. Further technical details and information on primer design can be found in the Protocol S1. To facilitate the design of primers, we generated primer sequences for the ∼190,000 previously described specific gRNA-targetable sequences [11] (Table S1).

### Generation of a Stably Expressing Cas9 Cell line (SEC-C)

The human codon-optimized Cas9 plus C-terminal nuclear localization signal (NLS) was PCR amplified from Plasmid 41815: (Addgene: 41815), adding a Flag tag to the C-terminus and NotI sites to the 5′ and 3′ ends with the primers 5′ GAGCGGCCGCCACCATGGACAAGAAGTACTCCATTGG 3′ and 5′ GTGCGGCCGCTCACTTGTCATCGTCGTCCTTGTAGTCCACCTTCCTCTTCTTCTTGGGGTCAG 3′. The PCR product was digested with NotI and ligated into the NotI site of pcDNA5 FRT/TO vector (Invitrogen) (Figure S2A). U2OS and HEK293 cells were grown in DMEM media supplemented with 10% (v/v) fetal bovine serum, 100 U/ml penicillin, 100 µg/ml streptomycin and 1% L-glutamate (GIBCO, Invitrogen). Cells stably expressing Cas9 were generated using the Flp-In T-REx system (Invitrogen) as described previously [13]. Briefly, 9 µg of POG44 recombinase (Invitrogen) and 1 µg of pcDNA5 FRT/TO Cas9-Flag were co-transfected into U2OS Flp-In T-REx and HEK293 Flp-In T-REx using GeneJuice (Millipore) and calcium chloride respectively. 48 hours after transfection, cells were selected with 100 µg/ml hygromycin and 10 µg/ml blasticidin. Expression of Cas9-Flag in U2OS SEC-C and HEK293 SEC-C was induced by the indicated tetracycline concentrations for 24 hours. The expression levels of Cas9-Flag 24 hours, after tetracycline induction versus 24 hours after transient transfection were compared by anti-Flag Western Blot. The expression of Cas9-Flag was higher in HEK293 SEC-C and comparable in U2OS SEC-C (Figure S2B).

### Immunoblotting

An anti-Flag M2 monoclonal antibody (F1804, Sigma-Aldrich) was used to detect Cas9-Flag expression followed by horseradish peroxidase conjugated anti-mouse IgG and SuperSignal detection kit (Pierce) according to the manufacturers protocol. Detection of FAN1 was carried out as described previously [13].

### Clonogenic survival analysis

U2OS cells were seeded in triplicate in 6 well plates and allowed to attach before treatment for 24 hours with Mitomycin C (MMC) (Duchefa). After 15 days, cells were washed, fixed and stained with crystal violet (Sigma). The number of colonies with>100 cells was counted. Results were normalized to plating efficiency. For each genotype, cell viability of untreated cells was defined as 100%. Data are represented as mean ± SEM from three independent experiments.

## Results and Discussion

The major refinements we made to the Cas9/CRISPR system are as follows: **1**. Generation of the sgRNA vector using a very efficient PCR-based method. **2**. Construction of cells stably expressing Cas9 (SEC-C) using the Invitrogen Flp-In T-REx U2OS cells. This system allows introduction of the gene of choice at FRT sites downstream of tetracycline-inducible elements. HEK293 cells in which the Cas9-Flag protein has been introduced have also been generated. SEC-C eliminates the need for low efficiency, bi-plasmid transfections; the sgRNA plasmid is simply transfected into these cells. **3**. Recombination-based flipping out of Cas9 from the SEC-C cells after gene disruption. This minimizes the risk of Cas9 causing inappropriate gene cleavage and re-generates a FRT site for complementation. **4**. Recombination-based complementation at the regenerated FRT sites of the gene that was disrupted in the first place. We used the Fan1 DNA repair gene as a control in these experiments. Cas9/CRISPR-mediated Fan1 disruption occurred at frequencies of around 29%, and resulted in the anticipated spectrum of genotoxin hypersensitivity, which was rescued by re-introduction of Fan1.

### Generation of the single guide (sg) RNA plasmid

**Figure 1:**
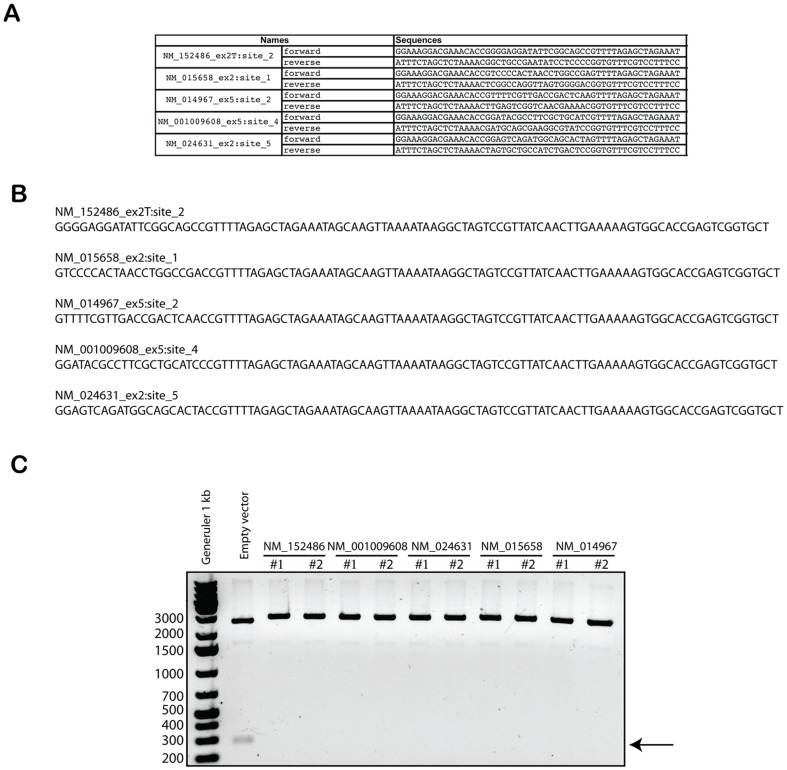
Fast PCR-based generation of sgRNA-encoding plasmids. (**A**) Table showing primers used to generate the sgRNA plasmids (pEsgRNA) for five different human ORFs using PCR-based insertion mutagenesis: NM_152486, NM_015658, NM_014967 (FAN1), NM_001009608 and NM_024631. (**B**) gRNA sequence transcribed from the U6 promoter. (**C**) sgRNA plasmids purified from bacteria were BamHI digested and separated using 1% agarose gel in TAE buffer. The arrow indicates the 300 bp BamH1 digest fragment.

Primers described in Figure 1A were used for PCR mutagenesis to introduce the sequence corresponding to the targeted gene into the pEsgRNA vector, generating the sgRNA indicated in the Figure 1B. The empty sgRNA plasmid has been designed so that when the plasmid is not mutated, BamHI digestion will generate a 300 bp fragment. As shown in Figure 1C, 2 colonies each were analyzed for 5 different sgRNAs generated and all lost the BamHI restriction site, demonstrating the efficiency of the method. Because this method is based on a single PCR reaction and avoids any ligation step, it is suitable for the generation of a large sgRNA library at a small cost and with minimal bench work. Additionally, some authors have used an sgRNA plasmid that includes a puromycin resistance gene. This cassette allows selection of cells that have been transfected by the sgRNA plasmid and thus increases the ratio of successfully targeted cells. Although puromycin selection marker has not been used in this study, we also have generated an sgRNA empty backbone with a puromycin resistance gene under an IRES promoter (DU46218). This plasmid can be used as a backbone for the presented PCR cloning method. It also allows selection of transfected cells with puromycin as described previously [14].

**Figure 2:**
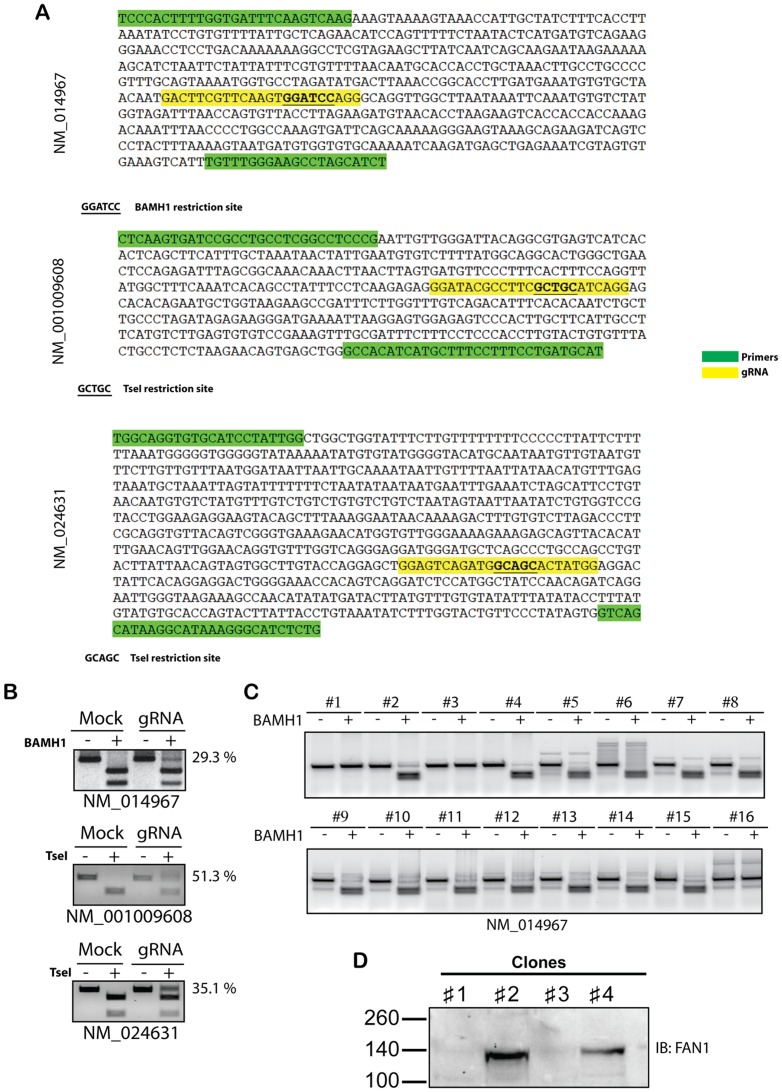
Analysis of gene targeting. (**A**) Sequences corresponding to the PCR products used to analyze the mutation of the targeted sites. Primer sequences and target sites are respectively highlighted in green and yellow. The restrictions sites used to analyze mutation of the targeted sequences are underlined and in bold. (**B**) PCR was performed using DNA isolated from a pool of U2OS SEC-C that had been transfected with the indicated sgRNA. PCR products have been purified and digested or not with the indicated enzymes. The indicated percentages represent the relative intensity of the top band of lane 4. Band intensities have been quantified using Image J software on three independent experiments (**C**) DNA isolated from single cell colonies was analyzed using the primers shown in Figure 2A. (**D**) Cell clones were analysed by western blot using an antibody against FAN1, the protein encoded by NM_014967.

### Generation of U2OS SEC-C and gene disruption

Traditionally, Cas9 and sgRNA are delivered in separate plasmids, or in one large plasmid that contains both sequences. The use of 2 plasmids, or of a large combined plasmid, decreases the efficiency of the transfection. In order to overcome this limitation, we decided to generate stable cell lines SEC-C that express a Flag tagged Cas9 protein under a tetracycline inducible promoter (Figure S2). We then targeted 3 different genes, corresponding to the NM-014967, NM-001009608 and NM_024631 cDNAs. These genes have been selected based on the presence of a restriction site (Figure 2A, bold underlined sequences) close to the sgRNA-targeted site (Figure 2A, yellow sequences). Because NHEJ repair of the double strand break often leads to deletions, the loss of this restriction site provides an easy method to screen clones. U2OS SEC-C were transfected independently with the 3 different sgRNAs and treated with tetracycline to induce the expression of the Cas9-Flag (Figure S2B) as indicated in the Protocol S1. 48 hours after the last sgRNA transfection, the set of primers shown in Figure 2A (green sequences) was used to amplify genomic DNA from the 3 targeted cells by PCR and analyzed the efficiency of the mutation by looking for the partial digestion of the amplified fragment (Figure 2B). NM_014967, NM_001009608 and NM_024631 amplified fragments lost the restriction site with 29.3%, 51.3% and 35.1% efficiency respectively. Isolation of single clones was carried out for the cells targeted against NM_014967 (FAN1) by serial dilution in 96 well plates. We then compared the efficiency of the SEC-C cells versus the bi-plasmid transfection protocol. As shown in Figure S3, the use of the SEC-C increased the efficiency and the consistency of allele mutation (34.2% +/−1.4 versus 17.8% +/−4.1). After growing the single clones for 2 weeks, we analyzed them using the same protocol (Figure 2C). Each allele of the clones 1, 2, 3 and 4 has been sequenced (Figure S4A) showing a stop codon in the alleles of clones 1 and 3 (Figure S4B). Surprisingly only one mutation has been identified in clone 1, indicating that both alleles present the same mutation, and two different mutations have been identified for the clone 3. Western blotting was used to confirm that clone 1 and 3 are KO for the targeted gene (Figure 2D).

### Cell complementation and phenotype

**Figure 3:**
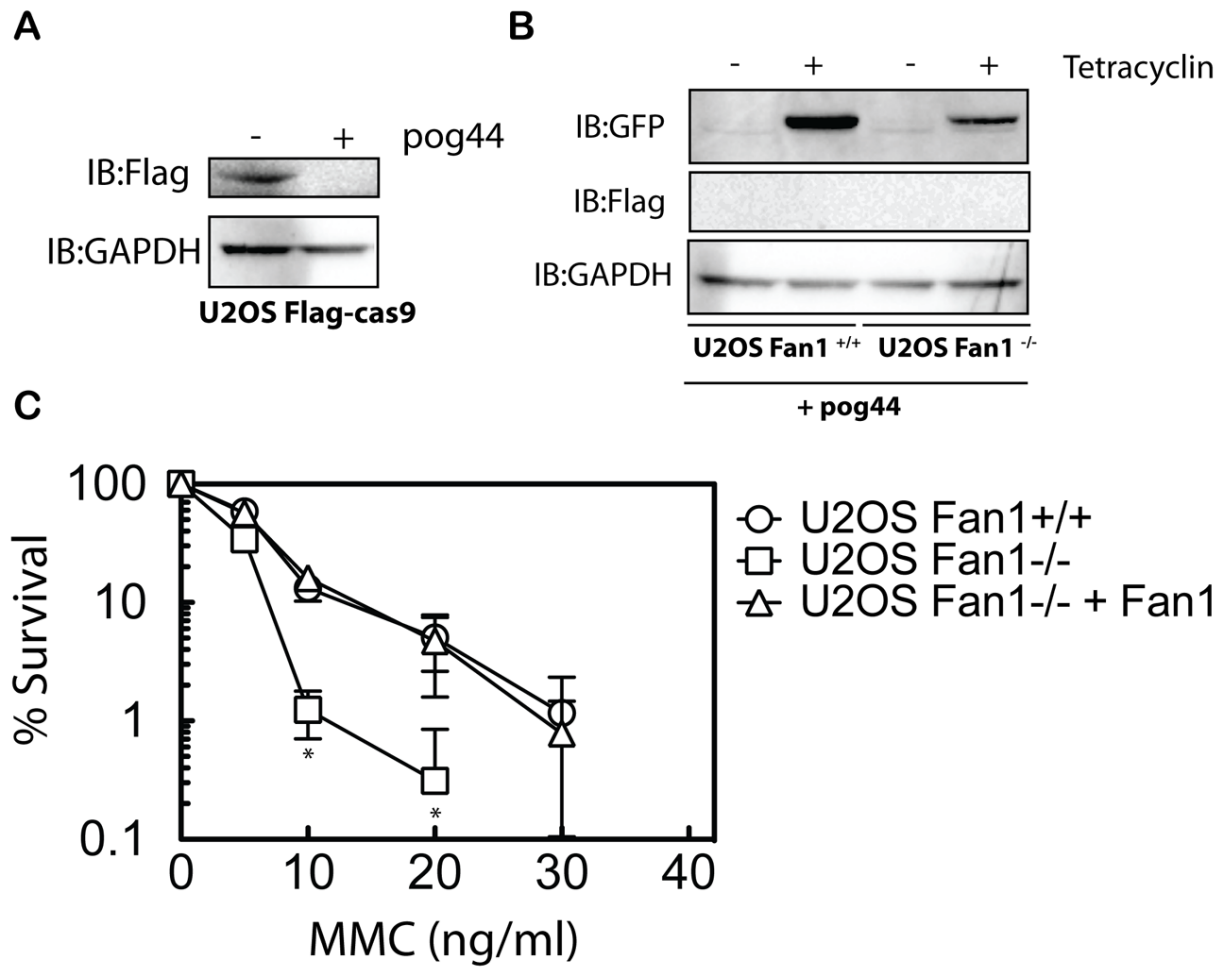
Recombination-based complementation of FAN1 knockout cells. (**A**) U2OS SEC-C FAN1^−/−^ cells were transfected or not with the plasmid pOG44 expressing the Flp recombinase. After 5 passages under zeocin selection, cell extracts were subjected to western blotting with anti-Flag antibodies to check for the elimination of the Cas9 ORF. (**B**) Lysates from Flp-In T-REx U2OS FAN1^−/−^ cells transfected or not with the plasmids pOG44 and pCDNA5-FRT-GFP-FAN1 were subjected to western blotting with the indicated antibodies. (**C**) Clonogenic survival analysis of the cells U2OS FAN1^+/+^ (clone 2) U2OS FAN1^−/−^ (Clone 1) and U2OS FAN1^−/−^ + FAN1 (Clone 1 complemented) after exposure to MMC. For each genotype, cell viability of untreated cells is defined as 100%. Data are represented as mean ± SEM, n =  Experimental significance was calculated using a unpaired T-test correct using Holm-Sidak method; *, p<0.01.

Because off-target effects have been described for the CRISPR system [8], [12], [15], [16], [17], it is necessary to complement the knock-out cells with the wild type allele to confirm any phenotype associated with the mutation. We decided to take advantage of the fact that in the cell line we generated, the Cas9 gene is flanked by two FRT-recombination sites (Figure S5A). This should allow the removal of the Cas9 cassette in a Flip recombinase dependent reaction, and the regeneration of a single intact FRT site. Thus, we transfected this cell line with the Flip recombinase-expressing vector, POG44 (Invitrogen). Cells were then selected with Zeocin for the loss of Cas9 (Figure S5A), also see Protocol S1). To verify the absence of Cas9 in these cells, they were then incubated with tetracycline as before. No expression of Flag tagged Cas9 was detected in the cells transfected with POG44, indicating a complete flip-out of the Cas9 cassette in these cells (Figure 3A). Finally these cells were co-transfected with a pCDNA5 vector containing a puromycin resistant cassette plus a GFP tagged version of FAN1 (DU45847), and the POG44 plasmid. Cells were then selected in medium containing puromycin and the tetracycline-inducible expression of GFP-FAN1 protein was confirmed by Western blotting using an anti-GFP antibody (Figure 3B). Fan1 is involved in DNA interstrand crosslink repair and cells lacking FAN1 have been shown to be hypersensitive to the genotoxin MMC. Thus, we analyzed the sensitivity of U2OS FAN1^+/+^, U2OS FAN1^−/−^ and U2OS FAN1^−/−^ complemented with GFP-FAN1 to MMC using a clonogenic survival assay. As shown in Figure 3C and Figure S5B, both clones of U2OS FAN1^−/−^ are sensitive to MMC as expected, and this sensitivity is completely rescued in the complemented U2OS FAN1^−/−^ + GFP-FAN1.

## Conclusions

The CRISPR genome editing method represents a significant advance in generating KO cell lines. However, some of the key steps can be problematic, and this, along with the potential for off target effects, deters many researchers from using the method. The described CRISPR method presents some advantages: The first change concerns the cloning of the sgRNA. Oligo annealing and ligation protocols, such as the one published by the Zhang lab [9], are widely used. The Zhang lab protocol consists of three steps (phosphorylation/annealing, digest/ligation and exonuclease treatment). On the other hand, the PCR insertion-based mutagenesis protocol reduces the number of steps to two (PCR and nuclease treatment) and approaches 100% efficiency. However, the use of a polymerase will possibly introduce mutations if used on a bigger plasmid. Thus we do not recommend the use of site-directed mutagenesis on larger sgRNA vectors such as the one including the Cas9 gene.

Secondly, the use of SEC-C is more consistent and efficient than transfection of the sgRNA and Cas9 in two different plasmids. Also, the use of the FRT sequence to flip out Cas9 and reintroduce the targeted gene, represents an easy and efficient way to complement the cells. This step is necessary to address possible off target effects and ensure the mutation and any identified phenotype are linked. The method is limited by the need to use the Flp-In T-REx system but it can be easily integrated into a range of cell lines (Flp-In T-REx Core Kit, Invitrogen) and to our knowledge U2OS, HEK293, Hela and HCT116 Flp-In T-REx have already been generated elsewhere. Since U2OS and HEK293 are well-established model cell lines we decided to stably express Cas9 in these cells. We are therefore confident that the described method will be highly valuable to many researchers.

Reference numbers (DU numbers) have been assigned to the plasmids used in this study. The plasmids (with sequences) are available as well as the U2OS SEC-C and HEK293 SEC-C cell lines at https://mrcppureagents.dundee.ac.uk/.

## Supporting Information

Figure S1 **Schematic diagram for the generation of the sgRNA plasmid by PCR-insertion mutagenesis.** Primers described in the Protocol S1 are used to amplify the sgRNA empty vector. To remove the template plasmid from the PCR reaction, PCR reaction is digested with DpnI, which only cleaves methylated DNA.
(TIF)

Figure S2 **Generation of stable cell lines expressing Cas9-Flag under a tetracycline inducible promoter (SEC-C).** (**A**) Schematic diagram of the plasmid used to generate U2OS and HEK293 SEC-C. (**B**) FLAG Western blot analysis from U2OS SEC-C and HEK293 SEC-C lysates after incubation with 0.1 or 1 µg/ml of tetracycline.
(TIF)

Figure S3 **Comparison of the mutation efficiency between SEC-C and double transfection.** (**A**) PCR was performed using DNA isolated from a pool of U2OS SEC-C that had been either mock transfected (lane 1 and 2) or transfected with sgRNA against NM_024631 (lane 3 and 4), and from U2OS cells that had been transfected with a mixture of 10 µg CAS9 Flag plasmid and sgRNA against NM_024631 (lane 5 and 6). PCR products were purified and digested or not with the indicated enzyme. (B) The relative intensity of the bands in lanes 4 and 6 was quantified in four independent experiments using Image Lab software. Values in the histogram represent the relative intensity of the top band per lane, expressed in percentage after background subtraction (lane 2, band 1 intensity). Data are represented as mean ± STDEV, n = 4 and p value  = 0.0016. Experimental significance was calculated using a paired T-test; **, p<0.01; (**C**) Table used to generate the histogram in (B).
(TIF)

Figure S4 **Sequence analysis of U2OS FAN1^−/−^ clones.** (**A**) Mutations identified from the PCR fragments generated from the indicated single cell colonies with the primers described in Figure 2. (**B**) Predicted translation of the Open Reading Frames corresponding to the sequences identified in (A)
(TIF)

Figure S5 **Complementation of the U2OS FAN1^−/−^ cells.** (A) Schematic diagram of the protocol used to flip-out the integrated Cas9-Flag from clones 1 and 2. (**B**) Clonogenic survival analysis of U2OS FAN1^+/+^ (clone 2) U2OS FAN1^−/−^ (Clone 3) and U2OS FAN1^−/−^ + FAN1 (Clone 3 complemented) cell lines after exposure to MMC. For each cell line, cell viability of untreated cells is defined as 100%. Data are represented as mean ± SEM, n = 3. Experimental significance was calculated using a unpaired T-test correct using Holm-Sidak method; *, p<0.01.
(TIF)

Table S1 **Primers needed for sgRNA generation.** List of sgRNA targeting human genome with the sequence of the forward primers needed to generate sgRNA.
(ZIP)

Protocol S1 **Details of the protocols used to generate a sgRNA and human KO cells.**
(DOCX)
