# Supplementary figures and images for: Improved Genome Editing in Human Cell Lines Using the CRISPR Method
Source: PLoS One. 2014 Oct 10;9(10):e109752. doi: 10.1371/journal.pone.0109752 (PMC4193831; doi:10.1371/journal.pone.0109752)

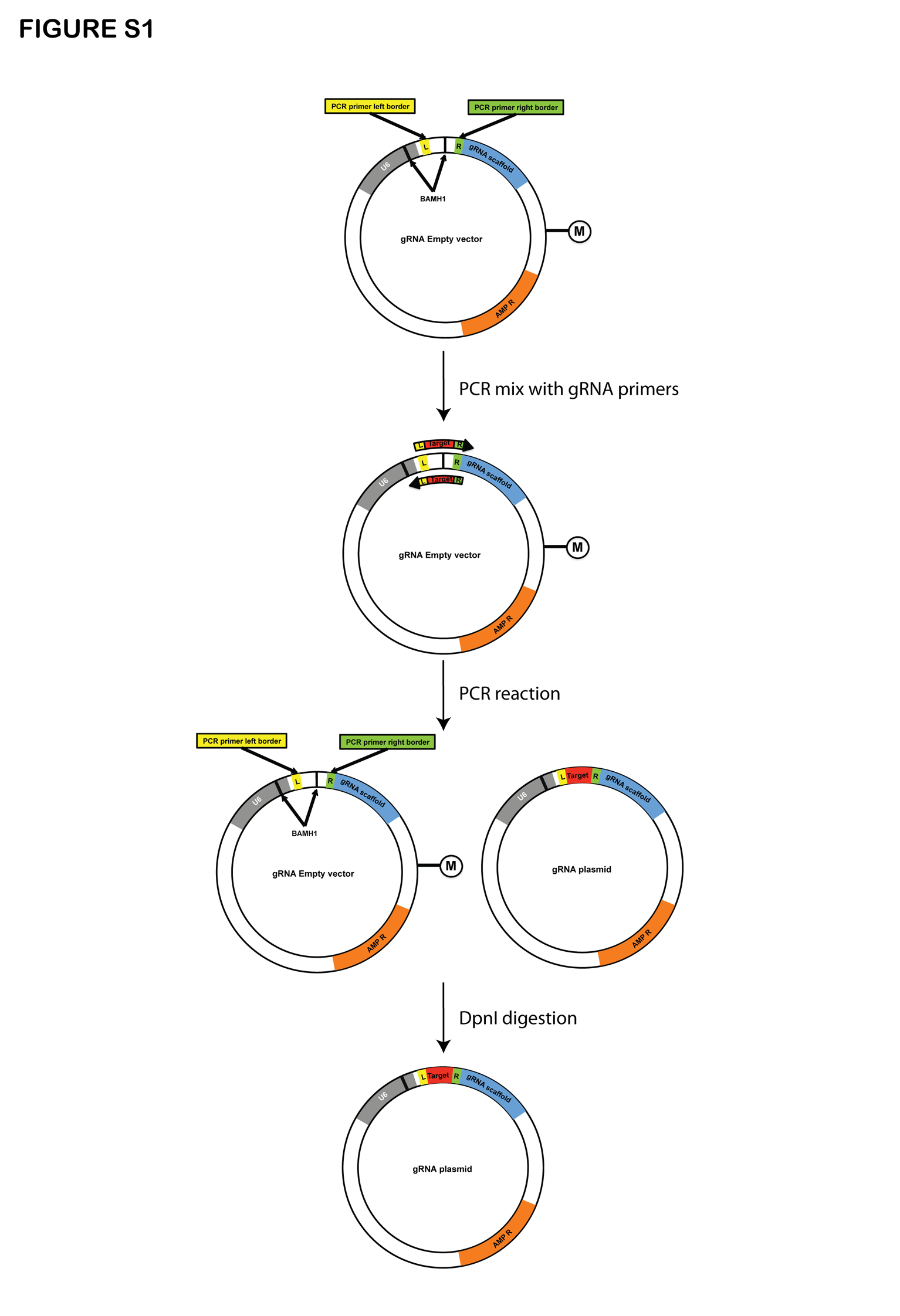

Supplement: Figure S1 — Schematic diagram for the generation of the sgRNA plasmid by PCR-insertion mutagenesis. Primers described in the Protocol S1 are used to amplify the sgRNA empty vector. To remove the template plasmid from the PCR reaction, PCR reaction is digested with DpnI, which only cleaves methylated DNA. (TIF) [file pone.0109752.s001.tif]

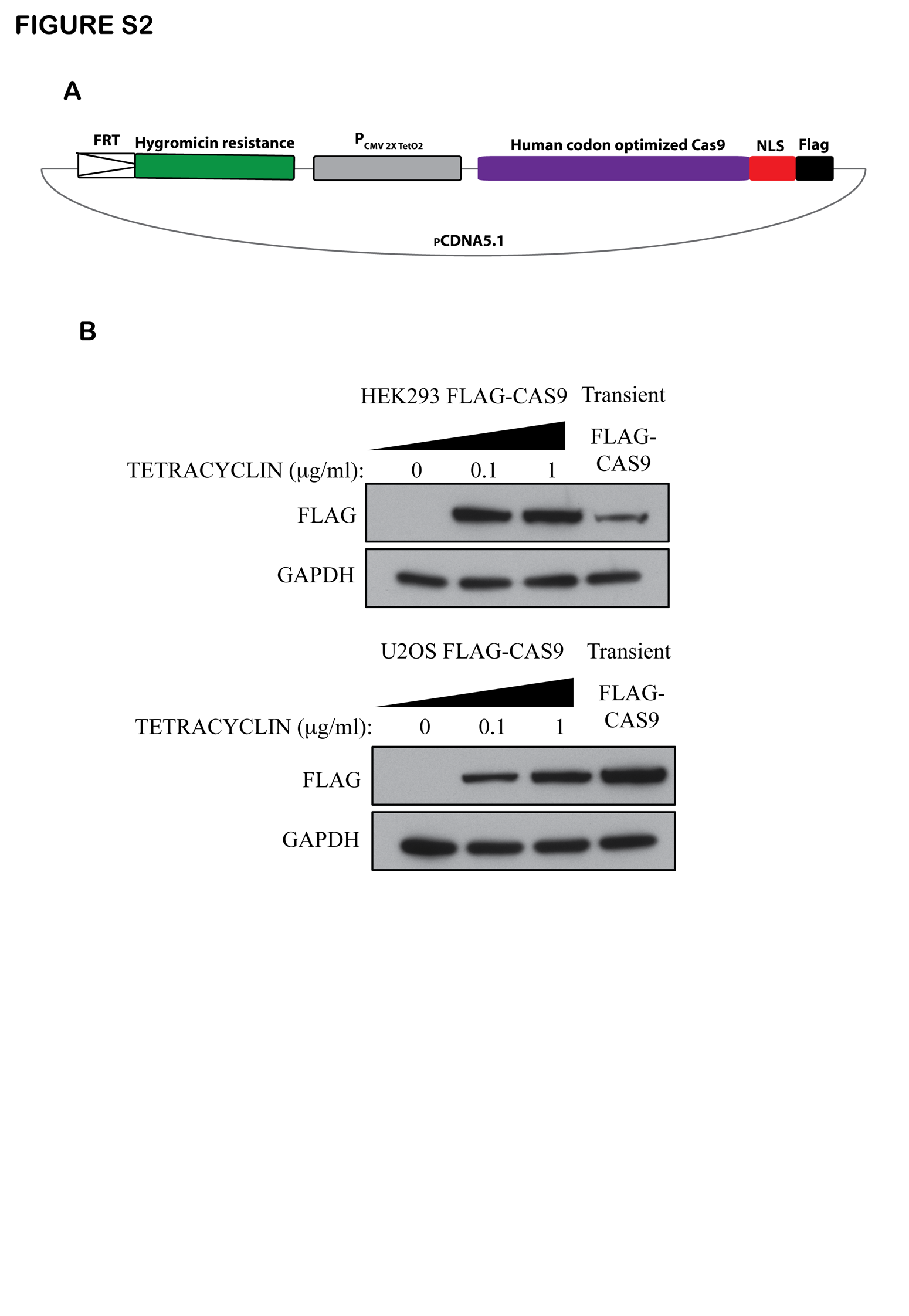

Supplement: Figure S2 — Generation of stable cell lines expressing Cas9-Flag under a tetracycline inducible promoter (SEC-C). (A) Schematic diagram of the plasmid used to generate U2OS and HEK293 SEC-C. (B) FLAG Western blot analysis from U2OS SEC-C and HEK293 SEC-C lysates after incubation with 0.1 or 1 µg/ml of tetracycline. (TIF) [file pone.0109752.s002.tif]

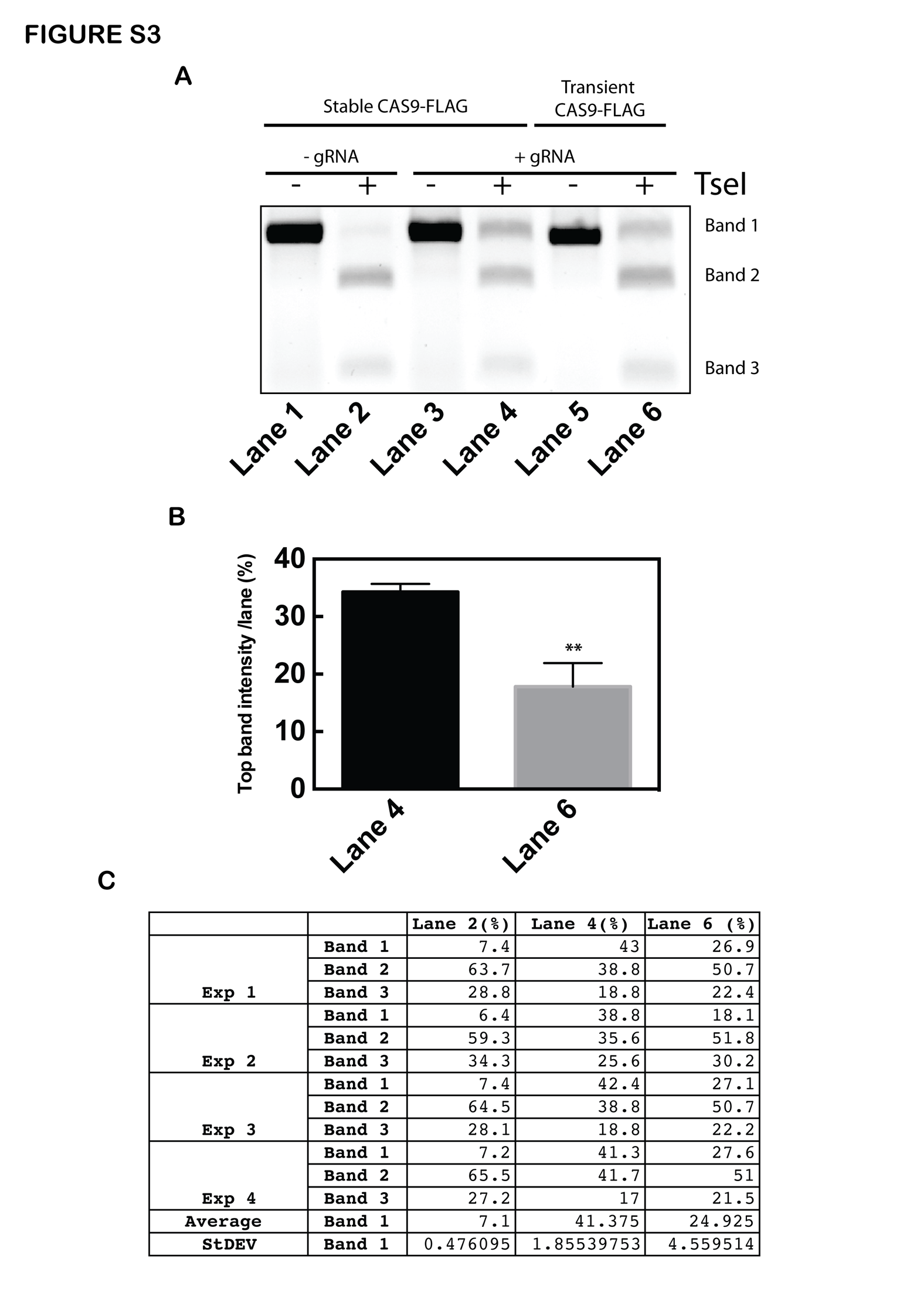

Supplement: Figure S3 — Comparison of the mutation efficiency between SEC-C and double transfection. (A) PCR was performed using DNA isolated from a pool of U2OS SEC-C that had been either mock transfected (lane 1 and 2) or transfected with sgRNA against NM_024631 (lane 3 and 4), and from U2OS cells that had been transfected with a mixture of 10 µg CAS9 Flag plasmid and sgRNA against NM_024631 (lane 5 and 6). PCR products were purified and digested or not with the indicated enzyme. (B) The relative intensity of the bands in lanes 4 and 6 was quantified in four independent experiments using Image Lab software. Values in the histogram represent the relative intensity of the top band per lane, expressed in percentage after background subtraction (lane 2, band 1 intensity). Data are represented as mean ± STDEV, n = 4 and p value = 0.0016. Experimental significance was calculated using a paired T-test; **, p<0.01; (C) Table used to generate the histogram in (B). (TIF) [file pone.0109752.s003.tif]

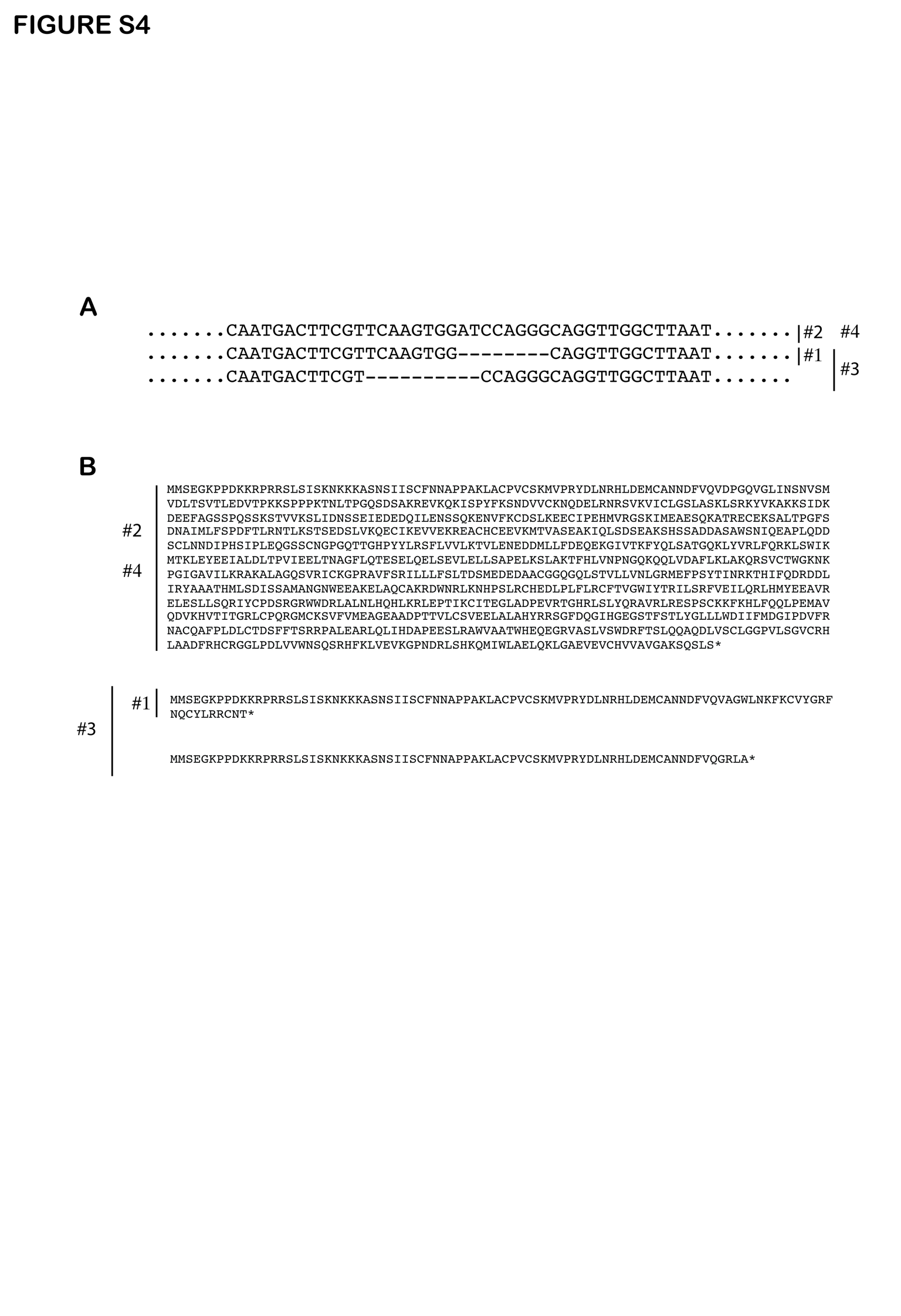

Supplement: Figure S4 — Sequence analysis of U2OS FAN1−/− clones. (A) Mutations identified from the PCR fragments generated from the indicated single cell colonies with the primers described in Figure 2. (B) Predicted translation of the Open Reading Frames corresponding to the sequences identified in (A) (TIF) [file pone.0109752.s004.tif]

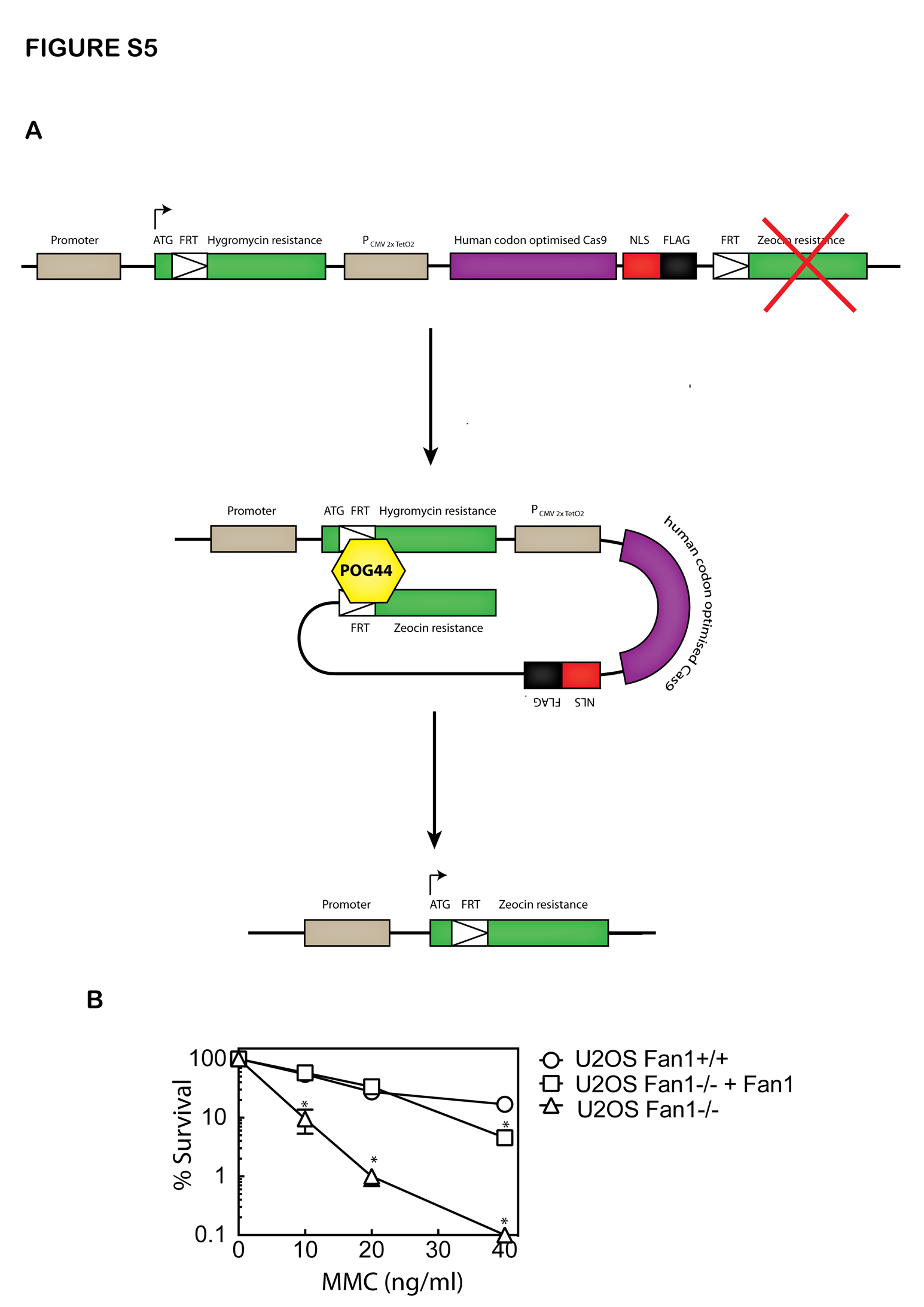

Supplement: Figure S5 — Complementation of the U2OS FAN1−/− cells. (A) Schematic diagram of the protocol used to flip-out the integrated Cas9-Flag from clones 1 and 2. (B) Clonogenic survival analysis of U2OS FAN1+/+ (clone 2) U2OS FAN1−/− (Clone 3) and U2OS FAN1−/− + FAN1 (Clone 3 complemented) cell lines after exposure to MMC. For each cell line, cell viability of untreated cells is defined as 100%. Data are represented as mean ± SEM, n = 3. Experimental significance was calculated using a unpaired T-test correct using Holm-Sidak method; *, p<0.01. (TIF) [file pone.0109752.s005.tif]
