# Supplementary material for: Improved Genome Editing in Human Cell Lines Using the CRISPR Method
Source: PLoS One. 2014 Oct 10;9(10):e109752. doi: 10.1371/journal.pone.0109752 (PMC4193831; doi:10.1371/journal.pone.0109752)
Supplement: Protocol S1 — Details of the protocols used to generate a sgRNA and human KO cells. (DOCX) [file pone.0109752.s007.docx]

**Reagent description**

**CRISPRs** (**C**lustered **R**egularly **I**nterspaced **S**hort **P**alindromic **R**epeats) are loci containing multiple short direct repeats that are found in the genomes of approximately 90% of sequenced archaea and 40% of sequenced bacteria. The CRISPR/Cas9 system can be implemented in mammalian cells by co-expressing the bacterial Cas9 nuclease along with the guide RNA. The method of cloning and expressing both of them is described below.

**1. Cas9-Flag expressing cell line (SEC-C):** This U2OS Flp-In T-REx cell line has been stably transfected with a Cas9-Flag vector under a tetracycline inducible promoter and a hygromycin resistance gene (vector design is indicated below). In order to express Cas9-Flag, cells growing under hygromycin (100 µg/ml) selection have to be treated with 1 µg/ml of tetracycline.

**2. DU46129, pEsgRNA:** This plasmid contains the chimeric guide RNA. A pair of oligonucleotides (design is indicated below and sequences are listed in the attached table) can be integrated into the guide RNA by a single PCR reaction. The oligonucleotides are designed based on the target site (20bp) and need to be flanked on the 5’ and 3’ by GGAAAGGACGAAACACC and GTTTTAGAGCTAGAAAT respectively. After PCR reaction the methylated backbone plasmid can be degraded by DPN1 digest. Clones can be analyzed by BAMH1 digest. Positive clones will be linearized and remaining backbones will produce an additional 300 bp fragment.

**gRNA cloning Protocol**

**DU46129, pEsgRNA**

In order to clone the target sequence into the pEsgRNA backbone, design two oligonucleotides as follows:

Primer1 5’-GGAAAGGACGAAACACCNNNNNNNNNNNNNNNNNNNNGTTTTAGAGCTAGAAAT-3’

Primer2 5’-ATTTCTAGCTCTAAAACNNNNNNNNNNNNNNNNNNNNGGTGTTTCGTCCTTTCC-3’

(List of primers available in attached table) 20Ns correspond to the targeted sequence

**gRNA cloning:**

1. PCR mix

KOD Hot start (Novogen Cat. No. 71086):

5 µl Buffer for KOD Hot Start DNA polymerase

3 µl 25 mM MgSO_4_

5 µl dNTPs (2 mM each)

X µl PCR Grade water

1.5 µl Primer1 10µM

1.5 µl Primer2 10µM

100 ng DU46129

1.5 µl DMSO

1µl KOD Hot Start Polymerase

----------------------------------------------------------------------------

50 µl Total reaction volume

2. PCR reaction

step 1 95°C 2 min

step 2 95°C 30 sec

step 3 53°C 45 sec

step 4 68°C 5 min 30 sec

return to step2 18 times

step 5 68°C 7 min

add 1ul DpnI (Fermentas FD1703)

step 6 37°C 30 min

step 7 65°C 20 min

3. Transformation

Transform thermo competent DH5α bacteria with 5 µl of the DpnI digested PCR product and select colonies with ampicillin.

step 1 0°C 10 min

step 2 42°C 2 min

step 3 0°C 2 min

4. BAMH1 digest

After maxiprep (qiagen QIAprep cat. No. 27104) digest 100 ng of plasmid with BamHI enzyme (Fermentas FD0054) and sequence positive clones by using M13 primers.

**Cell transfection**

**Cell transfection**

1. Reverse transfection

1 ml Optimem Medium (Gibco)

60 µl GeneJuice (Millipore 70967)

Vortex and incubate 10 min at RT

10 µg of gRNA plasmid

Mix and incubate 10 min at RT

Add mixture dropwise to the plate and add 2 10^6^ cell (U2OS_Cas9-Flag) on top of the transfection mix

2. Cas9-Flag induction

8 hours after transfection add 1 µg/ml of tetracycline to the cells.

3. 48 hours later

- Freeze down 1/3 of the plate

- Single cell colony them (in 96Well plates, use 100 µl per well of medium containing cells at a concentration of 10 cells/ml)

- Analyze mutation efficiency with the remaining 1/3 of the plate

**Cell Complementation**

**Cas9-Flag Flip-out**

1. Transfection

1 ml Optimem

60 µl GeneJuice (Millipore 70967)

Vortex and incubate 10 min at RT

10 µg of POG44 (DU13162)

Agitate and incubate 10 min at RT

Add the transfection mix on top of the cells.

2. Cell selection

After 2 days apply 5 days of 200 µg/ml of Zeocin (InvivoGen) on selected clones.

3. Cell transfection

Transfect the Cas9-Flag Flip-out cells with a pCDNA5 FRT/TO vector coding the gene of interest as described after.

(In this study we generate and use a pCDNA5 FRT/TO.GFP.Puro.DU (DU45825) empty vector to clone FAN1.)

1 ml Optimem

60 µl GeneJuice (Millipore 70967)

Vortex and incubate 10 min at RT

9 µg of POG44 (DU13162)

1 µg of your vector

Agitate and incubate 10 min at RT

Add the transfection mix on top of the cells.

4. Cell selection

After 2 days add the appropriate selection medium to cells.

(In this study we used 1.5 µg/ml puromycin)
